# Hematocrit Self-Testing in Patients with Polycythemia Vera and Other Hematological Conditions: Assessing the Accuracy of the StatStrip Xpress^®^ 2 LAC/Hb/Hct Device and User Opinion about the Device in Real-World Clinical Practice

**DOI:** 10.3390/jcm11144234

**Published:** 2022-07-21

**Authors:** Alicia Rovó, Claudia Baierlein-Leimbach, Cesare Medri, Ioannis Chanias, Loreen Errass, Theresa Fehr, Therese Triemer, Daphne B. McCarthy-Pontier, Thomas Lehmann

**Affiliations:** 1 Department of Hematology and Central Hematology Laboratory, Inselspital, Bern University Hospital, 3010 Bern, Switzerland; cesare.medri@insel.ch (C.M.); ioannis.chanias@insel.ch (I.C.); loreen.errass@insel.ch (L.E.); 2 Novartis Pharma Schweiz AG, 6343 Risch-Rotkreuz, Switzerland; claudia.baierlein@novartis.com (C.B.-L.); therese.triemer@novartis.com (T.T.); daphnepontier@hotmail.com (D.B.M.-P.); 3 Clinic for Medical Oncology and Hematology, Kantonsspital St. Gallen, 9000 St. Gallen, Switzerland; theresa.fehr@kssg.ch (T.F.); thomas.lehmann2@kssg.ch (T.L.)

**Keywords:** chronic myeloproliferative disorders, polycythemia vera, hematocrit, self-testing, quality of life, red cells, laboratory hematology

## Abstract

Maintaining hematocrit (Hct) levels below 45% can reduce morbidity and mortality in patients with polycythemia vera (PV). A device that patients can use to self-monitor Hct levels could enable timely interventions if Hct levels increase above 45%, and could improve quality of life (QoL). This study evaluated the accuracy of the StatStrip Xpress^®^ 2 LAC/Hb/Hct meter (Hb/Hct meter) when used by healthcare professionals (HCPs) or patients in clinical practice. Blood samples from 68 visits for 60 patients with PV or other hematological conditions were collected and analyzed by HCPs using a laboratory hematological analyzer, and by patients (self-test) and HCPs (professional test) using the Hb/Hct meter at two Swiss centers. Accuracy was assessed as the mean difference in readings between two users/methods (*mdiff*, 90% confidence interval; Spearman correlation [*r*]). The Hct values were similar between the professional test and analyzer (*n* = 66 comparisons, *mdiff* = 0.1% [−0.5 to 0.8]; *r* = 0.95, *p* < 0.001), the self-test and professional test (*n* = 62 comparisons, *mdiff* = −0.2% [−1.1 to 0.7]; *r* = 0.93, *p* < 0.001), and the self-test and analyzer (*n* = 63 comparisons, *mdiff* = 0.0% [−0.8 to 0.7]; *r* = 0.94, *p* < 0.001). The hemoglobin values across users/methods were also similar. Reporting their opinion on the Hb/Hct meter at visit 1, 100% of the patients found it easy to use, and 97% were willing to use it at home. Of the patients with PV, approximately 71% and 56%, respectively, stated that they would feel safer using a self-testing device, and that it would improve their QoL. These findings demonstrate the potential of the Hb/Hct meter for HCP and patient use in real-world settings.

## 1. Introduction

Polycythemia vera (PV) is a rare myeloproliferative neoplasm that is characterized by an increase in red blood cell mass [1], which can be measured according to hematocrit (Hct) or hemoglobin (Hb) levels [2] and, in many cases, an increase in white blood cell (WBC) and platelet counts [2]. Patients with PV are at increased risk of morbidity from thromboembolic events and cardiovascular death compared with age- and sex-matched people from the general population [3,4]; studies have shown that this risk is significantly higher in patients with Hct levels above 45% than in those with Hct levels below 45% [5]. Consequently, the treatment guidelines for the management of patients with PV emphasize the importance of maintaining Hct levels below 45% [6,7,8,9]. As such, it is necessary to monitor patients’ Hct levels so that treatment can be initiated in a timely manner [5,10,11]. In clinical practice, monitoring is typically carried out by healthcare professionals (HCPs) during routine outpatient visits. However, patients’ Hct levels can rise above 45% between visits, significantly raising their risk of morbidity and mortality. Increasing the frequency of the monitoring of Hct levels would ensure that patients can be treated rapidly, and so maintain their Hct below the 45% threshold for longer, along with providing “peace of mind” for patients and their treating HCPs.

In order to increase the frequency of monitoring of Hct levels, patients can visit outpatient clinics more regularly. However, outpatient visits can be associated with a substantial economic burden [12]. An alternative option would be for patients to regularly monitor their own Hct levels at home. Self-testing devices for home use are available and deliver good outcomes for other chronic medical conditions (e.g., blood glucose monitoring for patients with diabetes [13] or international normalized ratio monitoring in anticoagulated patients [14]). Additionally, while empowering patients to take more control of their disease management has been shown to lead to reduced consultations and improved disease outcomes and overall survival in oncology patients [15,16,17,18], there are currently no approved devices on the market that are suitable for patient use and that measure Hct directly (i.e., do not use Hb as an indirect measure of Hct).

To the best of our knowledge, the StatStrip Xpress^®^ 2 LAC/Hb/Hct meter (Nova Biomedical, Waltham, MA, USA; referred to as the Hb/Hct meter throughout this publication) is the only meter that provides direct measurements of both Hct and Hb levels from capillary (e.g., finger prick) blood samples while also being sufficiently user-friendly and affordable for patient self-testing at home. In Europe, the Hb/Hct meter has been recently approved for professional use but is not currently approved for patient use. The aim of this real-world proof-of-concept study was to investigate the suitability of the Hb/Hct meter as a self-testing device for patients with PV or other hematological conditions that require the regular monitoring of Hct and/or Hb values. Towards this goal, the current study assessed the accuracy of the Hb/Hct meter in measuring Hct and Hb values when used by patients or HCPs during routine outpatient visits at two Swiss centers, and collected their opinions on the Hb/Hct meter.

## 2. Materials and Methods

### 2.1. Study Design and Population

From 6 August 2020 to 16 April 2021, adult patients were recruited into this observational study at two Swiss hematology clinics. For inclusion, patients were required to have a confirmed PV diagnosis (World Health Organization 2016 criteria [19]), Hct < 35%, or Hct > 50%.

Patients were excluded if they were unwilling to have a finger-prick test and/or were unable to use the Hb/Hct meter without assistance. The patients’ Hct/Hb levels were monitored as part of routine practice, as advised by their HCP, and no additional visits were scheduled specifically for the study. Furthermore, the data obtained from the Hb/Hct meter were not considered by the HCPs in their management of the patient. The study was approved by the Ethics Committee of Canton Bern, Switzerland (study approval number 2020-00189).

The study aimed to achieve the following sampling quotas: at least 60% of the overall sample consisting of patients with PV, 30% consisting of patients with Hct < 35%, and 10% consisting of patients with Hct > 50%. These quotas were selected in order to assess the accuracy of the device in the measurement of Hct and Hb across a range of values that included extreme values (e.g., Hct < 35%, such as for patients with anemic conditions, or >50%, such as for patients with newly diagnosed PV). A minimum sample size of 60 readings per method was selected because it exceeded the minimum sample size of 40 recommended by the Clinical & Laboratory Standards Institute guidelines for the testing of a validated method [20], and because it provided sufficient statistical power to establish if any difference that was observed in the readings taken from the methods under comparison was significant.

### 2.2. StatStrip Xpress 2 LAC/Hb/Hct Meter (Hb/Hct Meter)

The Hb/Hct meter measures Hb and Hct from venous and capillary blood samples using an impedance method and test strips containing an electrode. Both the Hb and Hct values are measured directly from the sample, and are not calculated indirectly (i.e., the Hb values are not used as an indirect measure for the calculation of the Hct values). The range of values that can be measured with the device is 20–65% for Hct and 6.5–22.0 g/dL for Hb. The Hb/Hct meter does not measure other red blood cell parameters, such as the mean corpuscular volume.

### 2.3. Procedure

Prior to the start of the study, the HCPs at the participating clinics were trained by Nova Biomedical on how to perform the correct blood sampling using the Hb/Hct meter.

During the first patient visit (visit 1), the HCPs measured baseline Hct and Hb levels from venous blood samples using a laboratory hematological analyzer (referred to as “the analyzer”, Sysmex, Kobe, Japan) in order to assess the eligibility to participate. If eligibility was confirmed, the patients were informed about the study. If the patient was willing to take part in the study, signed informed consent was obtained. The participating patients were provided with the Hb/Hct meter, test strips, and instructions for use, and were asked to perform finger-prick blood sampling and Hb/Hct measurements on their own (self-test). The HCPs could answer essential questions from patients during the self-test, but they were not allowed to show or explain the procedure to the patients. The HCPs then repeated the finger-prick blood sampling and Hb/Hct measurement with the Hb/Hct meter (professional test). The patients were then asked to complete a questionnaire about use of the Hb/Hct meter (designed to take 15 min; Supplementary Information S1).

All of the patients attended visit 1. If follow-up visits were scheduled as part of their routine care, the blood measurements were repeated as described above. After visit 1, venous blood sampling and analysis with the analyzer could be performed before or after testing with the Hb/Hct meter. At the end of the study, the HCPs were asked to complete a 5-item questionnaire to assess their opinion of the device (Supplementary Information S2).

### 2.4. Endpoints

The primary endpoint was the accuracy of the Hct readings from the professional test versus the analyzer. Secondary endpoints included the accuracy of the Hct readings from the self-test versus the professional test, or the self-test versus the analyzer, the accuracy of Hb readings across different methods/users, the impact of blood cell counts on the accuracy of Hct readings from the professional test, and the opinions of patients and HCPs on the Hb/Hct meter.

### 2.5. Data Analysis

Continuous variables were summarized by the number of patients (*n*), mean, standard deviation (SD), and minimum, median, and maximum values. Categorical variables were summarized by their absolute and relative frequencies. Missing values were not replaced, and missing examinations were not repeated.

Accuracy was calculated as the mean difference (*mdiff*, 90% confidence interval [CI]) in absolute percentage points for Hct, or in g/L for Hb between the measurements obtained from different methods/users. The *mdiff* values were considered statistically equivalent if CI did not exceed 1% for Hct or 10 g/L for Hb (setting a significance level α = 0.05). The correlation coefficients (*r*) between the measurements from different methods/users were calculated using Spearman correlation. Bland and Altman plots were used to assess whether there was any variation in *mdiff* across the range of readings (e.g., at the upper and lower values). Spearman correlation (*r*) and regression coefficients were calculated from these plots. The possible impact of blood cell counts on the accuracy of the Hct readings from the professional test was assessed using Spearman correlation (*r*).

Measurements were excluded from analysis if they contained extreme values which did not align with routine medical validation, or if readings were missing. When assessing patient opinion on the Hb/Hct meter based on responses to the patient questionnaires, data were only analyzed from visit 1.

## 3. Results

### 3.1. Demographic and Clinical Characteristics

During the study period, blood samples were collected from a total of 68 visits across 60 patients. The mean age was 65.1 years, with 26.7% of the patients (16/60) ≥ 75 years of age, and 31.7% (19/60) were female. In total, 53.3% of the patients (32/60) had a diagnosis of PV. Of the remaining patients who did not have a PV diagnosis, 33.3% of the patients (20/60) had Hct values < 35%, and 13.3% of the patients (8/60) had Hct values > 50%. A summary of the baseline characteristics for the total cohort of patients is included in Table 1.

### 3.2. Summary of Measurements from the Blood Samples

Of the blood samples collected during the 68 visits, extreme values (i.e., values that were unexpectedly higher or lower than the historical records of the patient, and which did not align with routine medical validation) were observed with the Hb/Hct meter for four samples from three patients (Table 2). For two patients (Patient 1 and Patient 3), the measurement from the self-test was unusually low, while the measurement from the professional test was aligned with that from the analyzer. These unusual measurements were likely due to issues encountered when the patients performed the finger-prick sampling on themselves. In one patient (Patient 2), measurements from both the self-test and the professional test were unusually high, suggesting that the unusual measurements were likely due to technical issues with the Hb/Hct meter. Because the study protocol did not include the possibility of repeating the sampling and measurements in the event of unexpected results (e.g., after performing maintenance on the device if technical issues were suspected), these four measurements were excluded from the final analysis.

The four samples that were excluded from the Hct analysis due to their extreme Hct readings also presented similar discrepancies for the corresponding Hb readings (Table 2). The Hb readings from these four samples were therefore excluded from the final Hb analysis.

### 3.3. Accuracy of the Hct Readings from the Professional Test versus the Analyzer

For the comparison of the accuracy of the professional test with the analyzer, the final analysis included 66 professional tests versus the analyzer measurements (after one professional test was excluded as an outlier; *n* = 1/67 comparisons [1.49%]).

**Figure 1:**
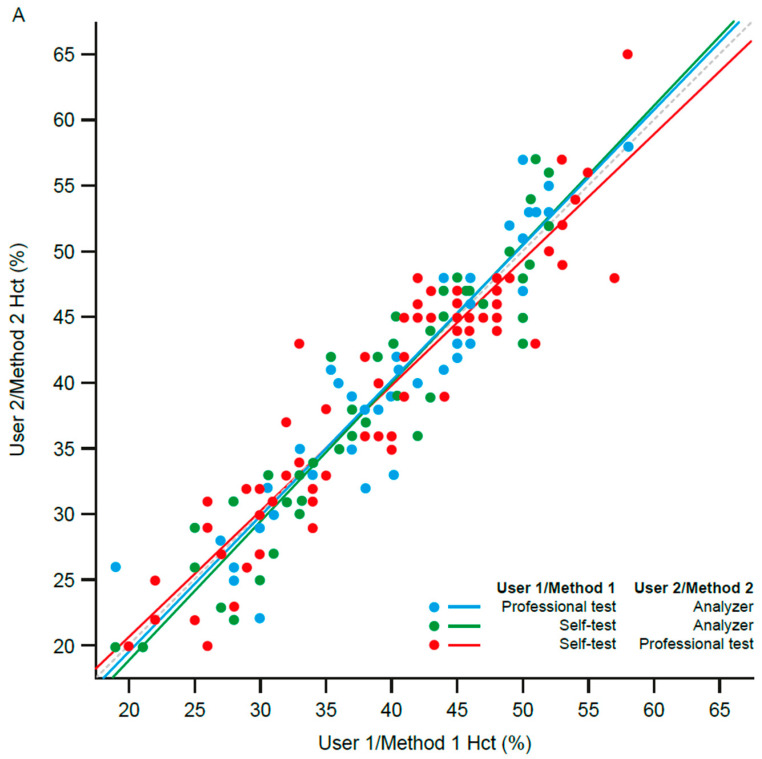

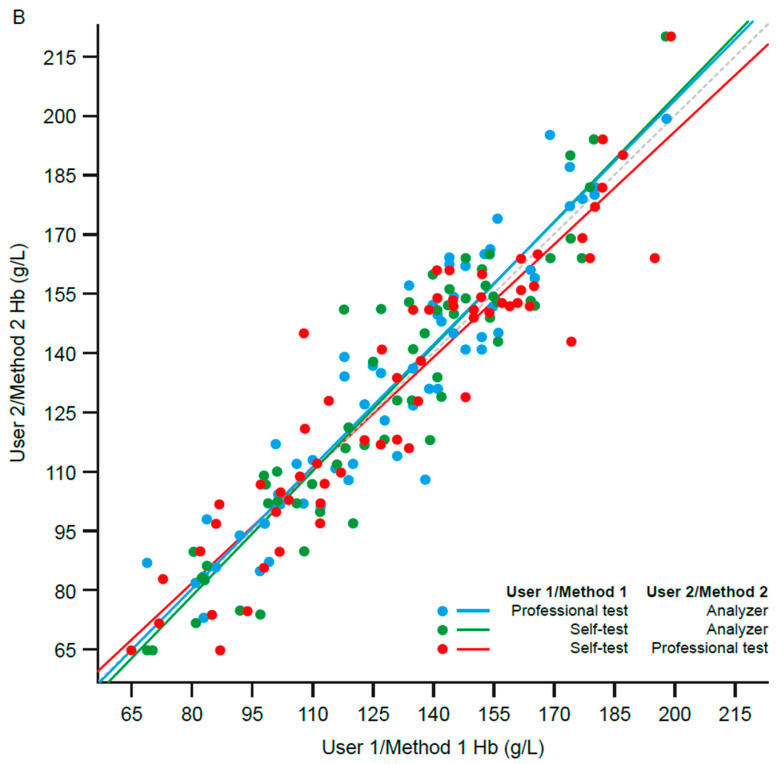
Accuracy of the Hct (**A**) and Hb (**B**) values collected via the StatStrip Xpress 2 LAC/Hb/Hct meter: (**A**) correlation between the Hct values obtained from the different analysis methods and users; (**B**) correlation between the Hb values obtained from the different analysis methods and users. A strong correlation denotes very similar results, indicating reliable readings. Hb, hemoglobin; Hct, hematocrit.

The values obtained from the professional test were, on average, nearly identical to those from the analyzer (*mdiff* = 0.1% [CI: −0.5 to 0.8]). There was also a strong positive correlation between the values obtained from the professional test and the values obtained from the analyzer (*r* = 0.95, *p* < 0.001; Figure 1A).

Bland Altman analysis (see Supplementary Information S1 for the methodological details) revealed a moderate correlation between the mean of the values obtained from the professional test and the analyzer, and their difference (*r* = 0.223), with a regression coefficient of 0.075.

There was no significant correlation between the accuracy of the professional test versus the analyzer and blood cell counts (in all cases *p* > 0.01). The correlation values are summarized in Table 3.

Furthermore, when the blood cell counts were stratified according to whether the readings were low, normal, or high, there was no significant impact on the accuracy of the professional test versus the analyzer (*p* > 0.05; Table 4)

### 3.4. Accuracy of the Hct Readings from the Self-Test versus the Professional Test

For the comparison of accuracy for the self-test with the professional test, the final analysis included 62 self-tests versus professional test measurements (after three self-tests and one professional test were excluded as outliers; *n* = 3/65 comparisons [4.62%]).

The values obtained from the self-test were, on average, similar to the values obtained from the professional test, but outside the threshold for statistical equivalence (*mdiff* = −0.2% [CI: −1.1 to 0.7]). There was also a strong positive correlation between the values obtained from the self-test and the values obtained from the professional test (*r* = 0.93, *p* < 0.001; Figure 1A).

Bland Altman analysis revealed no correlation between the mean of the values obtained from the self-test, the professional test, and their difference (*r* = 0.07), with a regression coefficient of 0.03.

### 3.5. Accuracy of the Hct Readings from the Self-Test versus the Analyzer

For the comparison of accuracy for the self-test with the analyzer, the final analysis included 63 self-tests versus analyzer measurements (after three self-tests were excluded as outliers; *n* = 3/66 comparisons [4.54%]).

The values obtained from the self-test were identical to the values obtained from the analyzer (*mdiff* = 0.0% [CI: −0.8 to 0.7]). There was also a strong positive correlation between the values obtained from the self-test and the values obtained from the analyzer (*r* = 0.94, *p* < 0.001; Figure 1A).

Bland Altman analysis revealed a moderate correlation between the mean of the values obtained from the self-test, the analyzer, and their difference (*r* = 0.31), with a regression coefficient of 0.11.

### 3.6. Accuracy of the Hct Readings from the Self-Test versus the Analyzer According to Patient Characteristics

When the data were stratified according to sex (male and female), the values obtained from the self-test were also nearly identical to the values obtained from the laboratory analyzer (*mdiff* = 0.0% [male] and *mdiff* = −0.1% [female]). When the data were stratified according to age (≤ 75 years and > 75 years), there was no significant difference in *mdiff* between the age groups (*mdiff* = 0.0% [CI: −0.8 to 0.7], *p* = 0.76), and there was no correlation between the age group and *mdiff* (*r* = 0.008). Finally, when the data were stratified by years since diagnosis for the subgroup of 32 patients with PV, there was no significant correlation between years since diagnosis and *mdiff* (*r* = −0.25, *p* > 0.05).

### 3.7. Accuracy of the Hb Measurements across the Analysis Methods

The same measurements that had extreme values for the Hct analysis were also omitted when assessing the accuracy of the Hb measurements between the users and methods.

Overall, the results were similar across the users and methods (Figure 1B). The professional test produced, on average, similar Hb readings to the analyzer (*n* = 66 comparisons; *mdiff* = 1.7 g/L [CI: −0.7 to 4.0]), and there was a strong positive correlation between the values obtained from the two methods (*r* = 0.93, *p* < 0.001). The Hb readings for the self-test versus the professional test were similar (*n* = 62 comparisons; *mdiff* = −0.7 g/L [CI: −3.9 to 2.5]), and there was a strong positive correlation for readings between the two users/methods (*r* = 0.93, *p* < 0.001). The self-test also produced similar Hb readings to the laboratory analyzer (*n* = 63 comparisons; *mdiff* = 1.1 g/L [CI: −1.8 to 4.1]), with the values between the two methods being strongly positively correlated (*r* = 0.93, *p* < 0.001).

Bland Altman analysis revealed a moderate correlation between the mean and the difference of the values obtained from the professional test and analyzer (*r* = 0.23), and the self-test and analyzer (*r* = 0.31), with a regression coefficient of 0.08 and 0.11, respectively. There was no correlation between the mean of the values obtained from the professional test, the self-test, and their difference (*r* = 0.06).

### 3.8. Patient and HCP Questionnaires about the Hb/Hct Meter

In order to assess patient opinion on the Hb/Hct meter, questionnaires that were completed at visit 1 were analyzed. In total, 60 patients completed the patient questionnaire at visit 1. Based on patient responses at their first visit, over 90% of the patients were satisfied or very satisfied with the Hb/Hct meter (*n* = 55/60; 92%), and all of the patients (*n* = 60/60; 100%) found it easy to use. Over 90% of patients understood how the test results were displayed on the screen (*n* = 55/60; 92%) and agreed with the statement that the test results were clear and easy to read on the screen (*n* = 58/60; 97%). Nearly all of the respondents (*n* = 58/60; 97%) were willing to start using the Hb/Hct meter at home, and 80% understood that they should eventually not make changes to their medication/therapy themselves based on the Hb/Hct meter test results.

For questions that focused on the opinion of patients with PV, responses were available from 34 patients from visit 1, indicating that two patients who did not have PV responded to these questions. Nevertheless, the majority of responses were from patients with PV. Based on responses from their first visit, 71% of the patients agreed that using a self-testing device would make them feel safer, and 56% of the patients agreed that it would have a positive effect on their QoL.

Eight HCPs completed the professional questionnaire at the end of the study. Nearly all of the HCPs (*n* = 7/8; 87.5%) were satisfied or very satisfied with their handling of the Hb/Hct meter, and half (*n* = 4/8; 50%) were satisfied or very satisfied with how the patients handled the device. Furthermore, over half of the HCPs (*n* = 5/8; 62.5%) agreed with the statement that they would trust patients to use the device at home with adequate guidance and practice; one HCP stated that they would want patients to receive thorough training, and the remaining two HCPs would use the device only for special patient cases, with thorough training.

## 4. Discussion

This proof-of-concept study assessed the accuracy of the StatStrip Xpress 2 LAC/Hb/Hct meter when used by patients or HCPs in clinical practice at two Swiss hematology centers, and collected patient and HCP opinions on the device. While the Hb/Hct meter is only approved for use by HCPs in Europe, this study demonstrates its potential suitability for use as an at-home self-testing device by patients with PV or other hematological conditions that require regular Hct and/or Hb monitoring.

In the present study, the Hb/Hct meter gave nearly identical Hct values to those from an analyzer when tests were conducted by HCPs, which is in line with a previous accuracy study of the Hb/Hct meter carried out by the manufacturer [21]. Similar Hct values were also obtained when the Hb/Hct meter was used by patients to when it was used by HCPs or compared to the analyzer. This demonstrates that the accuracy of the Hb/Hct meter is maintained when it is used by patients who have received minimal to no guidance from HCPs for conducting self-tests. Importantly, the accuracy of the Hb/Hct meter when used by patients was also not influenced by the time since diagnosis in the subset with PV, or by sex or age. This suggests that the device is reliable across a range of demographic and clinical characteristics, and that it is not influenced, for example, by a patient’s familiarity with technology (as might be the case for older versus younger patients), or by their condition. The Hb values were also similar for all of the comparisons between the analysis methods and users, and were all within the threshold for statistical equivalence (i.e., 10 g/L), suggesting that the Hb/Hct meter is also reliable for patient self-monitoring of Hb values.

Four blood samples from three patients, representing less than 3% of all of the samples taken with the Hb/Hct meter, were excluded from the final Hct and Hb analyses, under the assumption that they represented errors in blood sampling and/or technical issues with the Hb/Hct meter. The lack of previous studies investigating the use of a device such as the Hb/Hct meter to monitor Hct and Hb levels, as well as the fact that the protocol did not include instructions to repeat the tests in the event of unexpected results, meant that it was not possible to conclusively identify the reason for these extreme measurements. Furthermore, standardized training in the use of devices would likely influence the quality of the results from Hb/Hct measurements. Therefore, the limited training afforded to the patients in the present study might have also played a role in the extreme values observed in the two self-tests. Future study protocols should specify that unexpected measurements that are not aligned with the patient’s historical records should be repeated, in order to ascertain whether they represent accurate readings or reflect errors in sampling/technical issues with the Hb/Hct meter. Nonetheless, at least 95% of the measurements were included in the present analysis, suggesting that the use of a self-testing device is reliable in most cases.

Patients with PV can experience increased white blood cell and platelet counts, alongside increased red blood cell mass [1,2]. As such, this study aimed to include patients with both high and low blood cell counts. The accuracy of the Hb/Hct meter was additionally assessed according to blood cell counts based on the professional test versus the analyzer. The results suggest that neither high nor low blood cell counts had a significant impact on the device’s accuracy. However, these data are based on a very small number of samples with Hct values > 50% (*n* = 8), and the patient cohort did not include patients with extreme white blood cell (>200 × 10^9^/L) or platelet (>1000 × 10^9^/L) counts, which limits the conclusions that can be drawn.

Patient opinion about the Hb/Hct meter, as measured by the questionnaires at visit 1, was highly positive: over 90% of the patients found the device easy to use, and found it easy to read the results on its screen; nearly all of the patients stated that they would use the Hb/Hct meter at home. The patients with PV also largely agreed with the statement that the device would make them feel safer and might improve their QoL. The HCPs also felt the device was suitable for use by patients at home for self-testing, provided that the patients received adequate training. If the Hb/Hct device were to receive approval for patient use at home, it would be necessary to ensure that any training covered the importance of not modifying disease management without medical consultation. The patients should also be trained to repeat measurements if they obtain unusually high or low readings.

The Hb/Hct meter allows patients to test their own Hb and Hct levels regularly, between their standard visits to their HCP, in order to ensure more timely intervention should their Hct levels exceed 45%. However, it is important to note that the use of the Hb/Hct meter to monitor patients’ Hct and Hb levels at home would be intended to complement, and not replace, the monitoring of other crucial parameters in clinical practice that are essential in order to evaluate the overall health and the most suitable treatment approach in patients with PV or other hematological conditions. For example, alongside Hct and Hb levels, it could be important to consider whether a patient with PV has an iron deficiency, as well as their mean corpuscular volume (MCV) and zinc protoporphyrin (ZPP) concentration before deciding on the best approach to managing them if their Hct levels have risen above 45%. Another consideration when using the Hb/Hct meter is that some patients may experience severe hematoma/bleeding following the finger prick to collect a blood sample to test with the device. It is therefore important that HCPs carefully select patients to use the Hb/Hct meter who are unlikely to experience bleeding as an adverse event following blood sampling (e.g., through anticoagulation monitoring).

The main limitation of the present study was the relatively low number of the patients with extreme blood counts. This restricted the ability of the study to assess the accuracy of the device at more extreme ranges of Hct and Hb, as well as the impact of high or low white and red blood cell and platelet counts on the accuracy when measuring Hb and Hct. Another limitation relates to possible selection bias in the study cohort. This was because the HCPs may have only recruited patients who they felt would be able to perform self-testing with the Hb/Hct meter. Therefore, the patients who were too ill or who would require assistance to test may have been excluded from the study. However, it is important to note that this is likely a realistic reflection of how HCPs would identify which patients might be best suited to incorporate self-testing into the management of their condition.

## 5. Conclusions

In conclusion, based on results from a clinical setting, the present study highlights the potential suitability of the StatStrip Xpress 2 Hb/Hct meter for patients with any hematological conditions requiring the monitoring of Hct and Hb levels (e.g., the patients with PV, conditions in which patients experience anemia, conditions in which patients experience polycythemia) for incorporation into their routine care as a self-testing device for use at home. Benefits to patients may include increased QoL, improved clinical outcomes, and reduced morbidity and mortality through timely clinical interventions. In addition, regular self-monitoring may reduce the economic burden of certain hematological conditions and pressure on healthcare systems; for example, by decreasing the number of routine outpatient visits, which would in turn also reduce absences from work for clinical visits. Future research should evaluate the potential of the Hb/Hct meter in populations—beyond those investigated here—who may benefit from regular monitoring, as well as the long-term reliability of the Hb/Hct meter as a device for patient self-testing at home, the impact of its use on short- to medium-term clinical outcomes (e.g., whether its use is associated with a decrease in the number or frequency of clinical interventions, such as phlebotomies), and on the long-term impact of its use on clinical, economic and patient-reported outcomes.

## Figures and Tables

**Table 1:** Demographic and clinical characteristics of the patient cohort.

Baseline Characteristics	Total Cohort (*n* = 60)
**Age (years)**	
Mean (SD)	65.1 (12.8)
Median (IQR)	65.5 (58.0–76.0)
Range	28–86
**Sex, n (%)**	
Female	19 (31.7)
Male	41 (68.3)
**Diagnosis, n (%)**	
PV *	32 (53.3)
No PV	
Hct < 35%	20 (33.3)
Hct > 50%	8 (13.3)
**Blood cell counts, median (IQR)**	
White blood cells (10^9^/L)	7.6 (5.3–10.1)
Platelets (10^9^/L)	274.5 (188.0–382.0)
Erythrocytes (10^12^/L)	4.3 (3.5–5.5)

* According to WHO 2016 criteria. Hct, hematocrit; IQR, interquartile range; SD, standard deviation; PV, polycythemia vera; WHO, World Health Organization.

**Table 2:** Unexpected extreme measurements that were excluded from the analysis.

Patient ID	Hct Values (%)			Hb Values (g/L)		
	Analyzer	Professional Test	Self-Test	Analyzer	Professional Test	Self-Test
Patient 1	37	40	21	120	134	69
Patient 2	44	55	53	153	188	182
Patient 3	55	55	37	187	187	124

Hb, hemoglobin; Hct, hematocrit.

**Table 3:** Correlation between the accuracy of the professional test versus the analyzer and blood cell counts.

	Hct * (%)	WBC * (10^9^/L)	Platelets * (10^9^/L)	Erythrocytes * (10^12^/L)
Correlation coefficient ^†^	0.107	0.148	0.028	0.049
Significance (2-tailed)	0.390	0.234	0.824	0.696
Number of samples	66	66	66	66

* Measured using the analyzer. ^†^ Assessed according to the Spearman correlation coefficient. Hct, hematocrit; WBC, white blood cell.

**Table 4:** Accuracy of the professional test versus the analyzer (*mdiff*), stratified by the blood cell count values.

	WBC Count			Platelet Count			Erythrocyte Count		
	Low	Normal *	High	Low	Normal ^†^	High	Low	Normal ^‡^	High
*Mdiff,* % (professional test vs. analyzer)	−0.3	0.1	0.5	−0.5	0.6	−0.6	−0.1	0.2	0.5
90% CI mean	−3.0 to 2.4	−0.7 to 1.0	−1.7 to 2.6	−2.4 to 1.4	−0.4 to 1.6	−2.2 to 0.9	−1.4 to 1.3	−1.2 to 1.5	−0.7 to 1.6
Number of samples	10	43	13	10	41	15	23	29	14

* Normal WBC count = 4.5–11 × 10^9^/L. ^†^ Normal platelet count = 150–450 × 10^9^/L. ^‡^ Normal erythrocyte count = 4.0–5.5 × 10^12^/L. CI, confidence interval; *mdiff*, mean difference; WBC, white blood cell.

## Data Availability

Patient-level data underlying the findings of the present analysis are pseudo-anonymized, and are therefore not publicly available. This is in agreement with the consent forms signed by patients. The study protocol is presented in full in online Supplementary Information S3.

## Supplementary Materials

The following supporting information can be downloaded at: https://www.mdpi.com/article/10.3390/jcm11144234/s1. Supplementary Information S1: Patient questionnaire after the self-test with the StatStrip Xpress 2 LAC Hb/Hct meter; Supplementary Information S2: Healthcare professional questionnaire at the study’s conclusion; Supplementary Information S3: Study protocol [14,15,16,19,20,22,23,24].
