# Supplementary material for: Hematocrit Self-Testing in Patients with Polycythemia Vera and Other Hematological Conditions: Assessing the Accuracy of the StatStrip Xpress® 2 LAC/Hb/Hct Device and User Opinion about the Device in Real-World Clinical Practice
Source: J Clin Med. 2022 Jul 21;11(14):4234. doi: 10.3390/jcm11144234 (PMC9320629; doi:10.3390/jcm11144234)
Supplement: Supplementary file 1 [file jcm-11-04234-s001.zip › Rovo_et_al_HCT-HELP_Supplementary Information S1.pdf]

Supplementary Information S1

Patient questionnaire after self-test with the StatStrip Xpress 2 LAC Hb/Hct meter

Study site no.: ....

**Date:**	**Patient ID:**

**Questions on the meter**	**Response**		
1. Does the Nova StatStrip Xpress LAC/Hb/Hct device use capillary whole blood obtained from the fingertip?	**Yes**	**No**	**Don`t know**
2. Was the Hb and Hct monitor easy to use when performing a test?	**Yes**	**No**	**Don`t know**
3. When performing a Hb and Hct test, did you find it easy to apply the fingertip whole blood specimen to the test strip?	**Yes**	**No**	**Don`t know**
4. When testing was it easy to know when enough blood had been drawn into the test strip?	**Yes**	**No**	**Don`t know**
5. After reviewing the labeling were you able to understand how the Hb and Hct monitor works?	**Yes**	**No**	**Don`t know**
6. Would you feel comfortable using the Hb and Hct monitor to perform testing at home?	**Yes**	**No**	**Don`t know**
7. Did you find the lancets to be easy to use when lancing your fingertip to perform a test?	**Yes**	**No**	**Don`t know**
**Quick Reference Guide questions**			
8. After reviewing the Intended Use on page 1 of the Quick Reference Guide, do you feel you understand the intended use?	**Yes**	**No**	**Don`t know**
9. Did you find the Quick Reference Guide instructions to be easy to follow and understand?	**Yes**	**No**	**Don`t know**
10. Did you find the illustrations in the "quick start guide" clear and understandable?	**Yes**	**No**	**Don`t know**

**Hb and Hct test result questions**	
11. After performing testing, do you understand how the Hb and Hct test result is presented on the display screen?	**Yes** **No** **Don`t know**
12. Did you find the test results to be clear and easy to read on the monitor display?	**Yes** **No** **Don`t know**
13. It is planned to submit the StatStrip Xpress 2 Hb / Hct device for a home self-measurement to the authorities. If you can measure your blood values at home, do you know that you shouldn't make any changes to your medication or therapy yourself based on these Hb / Hct values?	**Yes** **No** **Don`t know**
14. Do you discuss the hemoglobin / hematocrit values with your doctor during regular consultations?	**Yes** **No** **Don`t know**

15. How did you get along with the StatStrip Xpress 2 Hb/Hct meter for self-measurement? Please tick smiley:

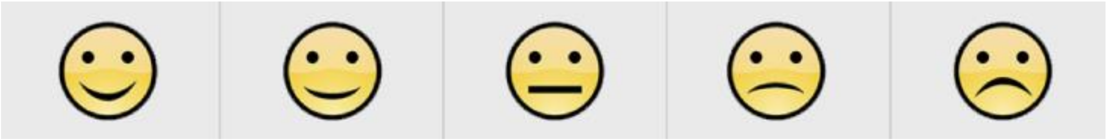

Very satisfied　Satisfied　Neutral　Not satisfied　Very dissatisfied

The following questions are only for patients with polycythemia vera (PV):

16. Since knowing your PV diagnosis, have you ever wondered about your hemoglobin or hematocrit levels?

- ☐ Yes
- ☐ No
- ☐ Don`t know

17. How often did you go to the doctor (e.g. family doctor) for a check-up of the PV outside of the hospital in 2019?

(An estimate is sufficient here)

- ☐ Approx. 12 times (corresponds to approx. every month)
- ☐ Approx. 6 times (every 2 months)

- ☐ Approx. 3 times (every 4 months)
- ☐ Approx. 2 times (every half year)
- ☐ Approx. 1 time a year
- ☐ Not at all in 2019

18. For a measuring device that you can use at home: how often would you measure your blood values (e.g. hematocrit) at home?

(An estimate is sufficient here)

- ☐ Several times a week
- ☐ 1 time a month
- ☐ 2 times a month
- ☐ 1 time a month
- ☐ Every 2 months
- ☐ Every 6 months

17. Would a measuring device at home help you to monitor your illness better and to feel safe?

- ☐ Yes
- ☐ No
- ☐ Don`t know

18. In your opinion, would a home measurement of your blood values (hematocrit or hemoglobin) have a positive effect on your quality of life or how you feel?

- ☐ Yes
- ☐ No
- ☐ Don`t know

19. Would you be ready to buy the StatStrip Xpress 2 Hb / Hct device for home measurements yourself?

- ☐ Yes
- ☐ No
- ☐ Don`t know
