# Supplementary material for: Hematocrit Self-Testing in Patients with Polycythemia Vera and Other Hematological Conditions: Assessing the Accuracy of the StatStrip Xpress® 2 LAC/Hb/Hct Device and User Opinion about the Device in Real-World Clinical Practice
Source: J Clin Med. 2022 Jul 21;11(14):4234. doi: 10.3390/jcm11144234 (PMC9320629; doi:10.3390/jcm11144234)
Supplement: Supplementary file 1 [file jcm-11-04234-s001.zip › Rovo_et_al_HCT-HELP_Supplementary Information S2.pdf]

Supplementary Information S2

Healthcare Professionals questionnaire at study conclusion

Study site: ....

**Date:**	**Study personnel (name, function):**

1. How did **you** get along with the StatStrip Xpress 2 Hb / Hct device when taking measurements on patients?

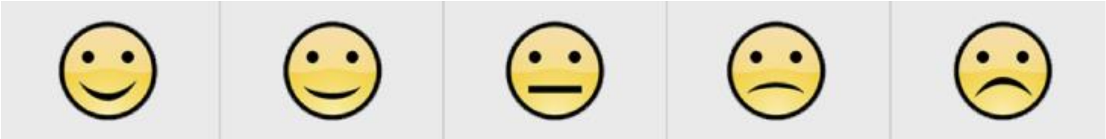

Very satisfied | Satisfied | Neutral | Not satisfied | Very dissatisfied

2. What was the most difficult thing for **you** when using the StatStrip Xpress 2 Hb / Hct device? (Please tick what comes closest to your answer.)

- ☐ Reading the manual (font size, images)
- ☐ Understanding the manual
- ☐ Opening the lancet (needle case)
- ☐ Finger prick (to obtain a blood drop)
- ☐ Filling the test strip with blood
- ☐ Operating the device (keyboard, inserting test strips, display)

3. How did the **patients** get along on average with the StatStrip Hb / Hct device during self-measurement?

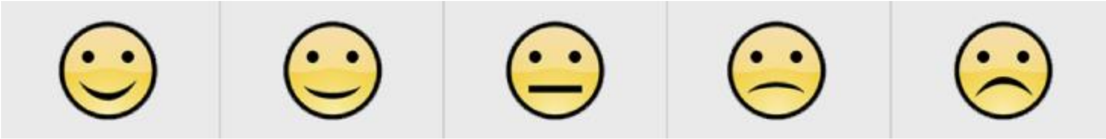

Very satisfied | Satisfied | Neutral | Not satisfied | Very dissatisfied

4. What do you think was the most difficult part for the **patient** using the StatStrip Xpress 2 Hb / Hct device? (Please tick what comes closest to your answer.)

- ☐ Reading the manual (font size, images)
- ☐ Understanding the manual
- ☐ Opening the lancet (needle case)
- ☐ Finger prick (to obtain a blood drop)
- ☐ Filling the test strip with blood
- ☐ Operating the device (keyboard, inserting test strips, display)

5. Would you trust **the patients** to use the measuring device at home (with appropriate instruction and practice)?

- ☐ Yes
- ☐ No
- ☐ Don`t know
