# Supplementary material for: Hematocrit Self-Testing in Patients with Polycythemia Vera and Other Hematological Conditions: Assessing the Accuracy of the StatStrip Xpress® 2 LAC/Hb/Hct Device and User Opinion about the Device in Real-World Clinical Practice
Source: J Clin Med. 2022 Jul 21;11(14):4234. doi: 10.3390/jcm11144234 (PMC9320629; doi:10.3390/jcm11144234)
Supplement: Supplementary file 1 [file jcm-11-04234-s001.zip › Rovo_et_al_HCT-HELP_Supplementary Information S3.pdf]

Research Project Protocol

CINC424BCH01R

HCT-HELP - Hematocrit adhoc self-testing in polycythemia vera patients

Proof of concept study

Authors: Alicia Rovo, Thomas Lehmann, Claudia Baierlein-Leimbach, Therese Triemer, Alexander Schulte, Daphne McCarthy

Document type: Research Project Protocol

Version number: 03

Release date: 02 June 2020

PROTOCOL SIGNATURE FORM

Study Title	HCT-HELP –<br>Hematocrit adhoc self-testing in polycythemia vera patients<br>Proof of concept study

The project leader (main center) and the investigator (at the local center/site) have approved the protocol version 03 (dated 02 June 2020), and confirm hereby to conduct the project according to the protocol, the Swiss legal requirements, the current version of the World Medical Association Declaration of Helsinki and the principles of Good Clinical Practice.

Project leader (lead center/site)

Site *[name and address of site]*

Insel Gruppe AG, Inselspital, Universitätsspital Bern, Freiburgstrasse, CH-3010 Bern
PD Dr. med. Alicia Rovó, Bereichsleiterin Klinische Hämatologie

Date: ____________________________ Signature: ______________________________

Local Project Leader at local center/site:

Site *[name and address of site]*

Kantonsspital St.Gallen, Rorschacher Strasse 95, CH-9007 St.Gallen
Dr. med. Thomas Lehmann, Klinik für Medizinische Onkologie und Hämatologie

Date: ____________________________ Signature: ______________________________

Table of contents

Table of contents 3
List of abbreviations 4
1 Responsible parties 5
2 Rationale and background 5
3 Study Objectives and endpoints 8
4 Research methods 10
4.1 Study design and study size 10
4.2 Setting 11
4.2.1 Patient population 11
4.2.2 Inclusion criteria 11
4.2.3 Exclusion criteria 12
4.2.4 Criteria for premature patient withdrawal 12
4.2.5 Initiation of study sites 12
4.2.6 Assessment types 13
4.3 Data sources and Visit schedule 15
4.4 Data management 17
4.5 Data analysis 18
4.5.1 Analysis set 18
4.5.2 Analysis of variables 18
4.5.3 Handling of missing values 18
4.6 Quality control - Data recording and document retention 19
5 Protection of human subjects 19
5.1 Informed consent procedures 19
6 Management and reporting of adverse events/adverse reactions 19
7 Plans of disseminating and communicating study results 20
8 References 20

List of abbreviations

CRF	Case Report/Record Form
CRO	Clinical Research Organization
EC	Ethics Committee
ECOG	Eastern Cooperative Oncology Group
Hb	Hemoglobin
Hct	Hematocrit
ICF	Informed Consent Form
ICMJE	International Committee of Medical Journal Editors
INR	International Normalized Ratio
JAK	Janus Associated Kinase
MedDRA	Medical Dictionary For Regulatory Activities
PHI	Protected Health Information
POC	point-of-care
PV	Polycythemia vera
RBC	Red Blood Cell
WBC	White Blood Cell
WHO	World Health organization

1 Responsible parties

Table 1-1 Main responsible parties

Sponsor	Inselspital Bern
Principal Investigator	PD Dr. med. Alicia Rovo (Leitende Ärztin / Stv. Chefärztin, Bereichsleiterin Klinische Hämatologie, Inselspital Bern)
Investigator of the second study site	Dr. med. Thomas Lehmann (Leitender Arzt, Klinik für Medizinische Onkologie und Hämatologie, Kantonsspital St. Gallen)
Protocol co-authors	Dr. Claudia Baierlein-Leimbach (field-based Medical Advisor, Novartis Pharma Schweiz AG)<br>Dr. Therese Triemer (Medical Advisor, Novartis Pharma Schweiz AG)<br>Dr. Daphne McCarthy (Therapeutic Area Head, Novartis Pharma Schweiz AG)<br>PD Dr. Alexander Schulte (Medical Scientific Liaison, Novartis Pharma GmbH, Deutschland)

2 Rationale and background

Polycythemia vera (PV) is a Philadelphia chromosome-negative MyeloProliferative Neoplasm (MPN) associated with dysregulated Janus Associated Kinase (JAK) signaling, specifically JAK1 and JAK2. Polycythemia vera is characterized by clonal stem cell proliferation of the erythroid, myeloid, and megakaryocytic lines, and while its predominant characteristic is an increase in red cell mass, increased white blood cell (WBC) and platelet counts are common. During the chronic phase, the proliferation of erythropoiesis is in the foreground and determines the clinical picture.

Patients with polycythemia vera have increased risks of morbidity and mortality relative to comparable subjects in the general population (e.g., same sex/age), often resulting from thromboembolic events or fibrotic/leukemic disease transformation. The disease carries a substantial symptom burden, most commonly pruritus and fatigue, affecting patient quality of life. Additionally 30-40% of polycythemia vera patients experience splenomegaly (Berk et al 1986, Stuart et al 2004, Mesa et al 2007, and Barosi et al 2013).

Diagnosis of polycythemia vera can be confirmed by the following criteria (Arber et al 2016, Barbui et al 2018):

Major Criteria:

1. Increased Hemoglobin (Hb) (>16.5 g/dL in men, >16.0 g/dL in women) and/or Hematocrit (Hct) (>49% in men, >48% in women) and/or increased red cell mass (RCM) (more than 25% above mean normal predicted value)
2. Bone marrow (BM) biopsy showing hypercellularity for age with trilineage growth (panmyelosis) including prominent erythroid, granulocytic, and megakaryocytic proliferation with pleomorphic, mature megakaryocytes (differences in size)

3. Presence of JAK2 V617F or JAK2 exon 12 mutation

Minor criterion:

- Subnormal serum erythropoietin level

Diagnosis of PV requires meeting either all 3 major criteria, or the first 2 major criteria and the minor criterion.*

*(Criterion number 2 (BM biopsy) may not be required in cases with sustained absolute erythrocytosis: hemoglobin levels >18.5 g/dL in men (hematocrit, 55.5%) or >16.5 g/dL in women (hematocrit, 49.5%) if major criterion 3 and the minor criterion are present. However, initial myelofibrosis (present in up to 20% of patients) can only be detected by performing a BM biopsy; this finding may predict a more rapid progression to overt myelofibrosis (post-PV MF).)

Patients which are diagnosed with polycythemia vera require timely interventions and treatments. Current treatment in PV has not affected the natural history of the disease in regards to overall, leukemia-free or myelofibrosis-free survival, but thrombosis-free survival has been positively affected by treatment with phlebotomy (Marchioli et al 2013), aspirin (Landolfi et al. 2004) and cytoreductive drugs e.g. hydroxyurea or Ruxolitinib (Tefferi and Barbui 2017).

In addition, it has been shown that maintaining a hematocrit target of 45 to 50% was associated with four times the rate of death from cardiovascular causes or major thrombosis, as was maintaining a hematocrit target of less than 45% (Marchioli et al 2013). It is therefore important to monitor Hct at regular intervals and maintain the value below 45% in order to keep the risk for thromboembolic events low.

Due to the chronic condition blood values of PV patients are monitored regularly during follow-up visits in the clinical routine. So far, the PV patientsp are scheduled to come to the clinic/office because of their unknown actual health status and Hct value.

Nowadays many chronic medical conditions can be controlled by patients at home (e.g. blood glucose self-monitoring in diabetics (Schnell et al. 2011) or for in-home INR (International Normalized Ratio) monitoring in anticoagulated patients (da Silva Saraiva et al. 2016)). In all these cases, there is a need for an instrument/device that can perform the measurement in a simple and reliable way by the patient at home. In general, self-control carried out by the patient in any clinical condition requires training and greater understanding of his/her disease and might change patients' attitudes towards their own health care.

Although Hct self-testing at home is not yet part of the current routine medical practice it might provide the following benefits:

- additional insights in Hct values and the trend between the doctors’ visits due to more frequent monitoring,
- enhance the exchange between patient and physician regarding treatment needs,
- determine an individual patients visit schedule supported by self-measured Hct values and proactively feedback from the patient to the treating physician,

- and most importantly resulting in more precisely treatments to maintain Hct < 45% and therefore keep the time spent in this target range as long as possible.

Empowering the patient to self-test their blood values might also have implications for the patient's quality of life and disease monitoring optimization due the possibility of fewer medical visits for hematocrit control, and minimize loss of working days (socio-economic aspect), and mainly improvement in disease management with more precision in the treatment. Ultimately, it will be desirable to enable PV patients in taking more control of their disease management with potential positive effects seen for example in malignant diseases resulting in an improved overall survival (Basch et al. 2016, Basch et al. 2017, Denis et al. 2017, Denis et al. 2019).

Although there are point-of-care (POC) devices for Hct/Hb measurement available in the European market, those are approved for medical professional use only and not approved for patient self-use. The existing POC devices for Hct/Hb are mainly in clinical use for screening of blood donors in blood banks or in private practices for monitoring purposes.

During our research for a potential POC device that directly measures Hct (not calculating it from the underlying Hb value) we identified only one suitable device in the market named "StatStrip Hb/Hct" manufactured by Nova Biomedical Corporation. The StatStrip Hb/Hct device measures hemoglobin and hematocrit from whole blood samples (venous and capillary) using the impedance method and test strips containing an electrode. The blood sample is usually obtained by finger pricking and the blood drop is absorbed by the test strip. The StatStrip Hc/Hct device can measure within following ranges: Hb: 6.5–22 g/dL (= 4.0–13.7 mmol/l); Hct: 20%–65%.

There is information available concerning the accuracy of the StatStrip Hb/Hct device technology in measuring Hct in normal references, however there is few data measuring high Hct (>50%) (e.g. in the case for newly diagnosed PV patients) or low Hct (<35%) values (e.g. anemic conditions) from patients (personal communication with Nova Biomedical and StatStrip® Hemoglobin and Hematocrit Meter System, Analytical Performance Testing, Nova Biomedical Corporation, Version Jan 2018).

Nova Biomedical Corporation has another device under development (not yet market authorized), named StatStrip Xpress 2 Hb/Hct, with the same underlying measurement technology like the already available StatStrip Hb/Hct device. The advantage of the Xpress version is a smaller size, less buttons and less included software. The StatStrip Xpress Hb/Hct device would therefore be more suitable for patient self-testing. Nova Biomedical provides those Xpress devices for the current study for research purposes.

This proof of concept study aims to proof the accuracy of the StatStrip Xpress 2 Hb/Hct device in measuring Hb/Hct values in a more real-world setting in two Swiss clinics (that actually monitor PV patients) and to evaluate the feasibility for patient self-testing. The "proof of concept study" is the first step of a process which aim to reach the possibility to evaluate the option of Hct self-testing in PV patients at home. Depending on the results of the present "proof

of concept study“, it is planned to set-up another study to extend the above research questions in a larger trial to test feasibility of Hct self-testing in PV patients at home.

Based on the «Verordnung über die Humanforschung mit Ausnahme der klinischen Versuche (810.301) (Stand am 24. April 2018)» des HFV (Humanforschungsverordnung) our study concept is categorized as research project. In article 7 (1, 3) of this directive a research project with minimal risk and strains (e.g. surveys and observations, periphery venous and capillary blood sampling) is classified as a research project of category A.

3 Study Objectives and endpoints

**Primary objective:**<br>to test the StatStrip Xpress 2 Hb/Hct device for accuracy in the clinical routine in measuring **Hct** from capillary blood via study personnel in comparison to value obtained from venous blood with the standard hematology analyzer.	**Primary endpoint:**<br>Mean difference (bias) in percentage points comparing Hct (Professional prick) vs. Hct (hematology analyzer)
**Secondary objectives:**	**Secondary endpoints:**
to test the StatStrip Xpress 2 Hb/Hct device for accuracy in the clinical routine in measuring **Hct** from capillary blood via patient self-testing in comparison to value obtained from venous blood with the standard hematology analyzer.	Mean difference (bias) in percentage points comparing Hct (patient self-prick) vs. Hct (hematology analyzer)
to test the StatStrip Xpress 2 Hb/Hct device for accuracy in the clinical routine in measuring **Hct** from capillary blood via patient self-testing in comparison to value obtained from the professional prick.	Mean difference (bias) in percentage points comparing Hct (patient self-prick) vs. Hct (professional prick)
to test the StatStrip Xpress 2 Hb/Hct device for accuracy in the clinical routine in measuring **Hb** from capillary blood via study personnel in comparison to value obtained from venous blood with the standard hematology analyzer.	Mean difference (bias) in g/dL comparing<br>Hb (professional prick) vs. Hb (hematology analyzer)
to test the StatStrip Xpress 2 Hb/Hct device for accuracy in the clinical routine in	Mean difference (bias) in g/dL comparing

measuring **Hb** from capillary blood via patient self-testing in comparison to value obtained from venous blood with the standard hematology analyzer.	Hb (patient self-prick) vs. Hb (hematology analyzer)
to test the StatStrip Xpress 2 Hb/Hct device for accuracy in the clinical routine in measuring **Hb** from capillary blood via patient self-testing in comparison to value obtained from the professional prick.	Mean difference (bias) in g/dL comparing<br>Hb (patient self-prick) vs. Hb (professional prick)
To evaluate the possible impact of a high blood cell count on the accuracy of the StatStrip Xpress 2 Hb/Hct device	Correlation of Hct values obtained by professional prick with RBC, WBC and platelet count
To evaluate the opinion of the patient on the handiness of the device	Questionnaire for patients
To evaluate the opinion of the study personnel on the feasibility of the device	Questionnaire for study personnel
To characterize the possible need of Hct home measuring in the population of PV patients	- patient questionnaire<br>- date of diagnosis<br>- age<br>- ECOG performance status<br>- actual treatments and treatment history of the last 12 months (phlebotomy frequency, cytoreductive therapy, anticoagulation therapy)<br>- blood parameters (Hb, Hct)<br>- number of hematological visits (at study site) of the last 12 months in which Hct value was above 45%<br>- number of visits for routine monitoring of PV in the last 12 months outside of the study site

The results of the current proof of concept study (depending on the outcome) might be used to set-up a bigger study in the future investigating the following potential aspects:

- to evaluate the accuracy of the Hct self-testing by the patients at home in long-term
- to gather insight into Hct values between doctor consultations based on home measuring

- to investigate the potential positive impact of self-test Hct monitoring at home on the patients visit schedule, impact on quality of life and impact on health care systems (e.g. payers)

4 Research methods

4.1 Study design and study size

This is a research project in an adult population (≥ 18 years old) of men and women who have been diagnosed with a condition that leads to check Hct and Hb values in clinical routine obtained by the standard hematology analyzer. The primary objective is to test the accuracy of Hct measurement with the StatStrip Xpress 2 Hb/Hct device compared to the hematology analyzer. The treatment of the patient will follow the clinical routine based on the values obtained by the hematology analyzer and is not impaired by the outcome or values of the StatStrip Xpress 2 Hb/Hct device. As this is an observational study, no formal hypothesis will be tested.

According to the 2018 guideline from the Clinical & Laboratory Standards Institute (CLSI), a sample size of 40 are adequate to test an already validated method (*Measurement Procedure Comparison and Bias Estimation Using Patient Samples – 3rd Edition. EP9c;* chapter 7.1). In this case the market authorized StatStrip Hb/Hct device with its impedance method was already validated regarding bias, imprecision, and linearity (StatStrip® Hemoglobin and Hematocrit Meter System, Analytical Performance Testing, Nova Biomedical Corporation, Version Jan 2018).

However the current study includes 60 measuring points to provide a power of 84% in a one sample t-test to test whether mean difference significantly does not exceed 1 percentage point. This is under the assumption of a standard deviation of 2.5 percentage points on a significance level of alpha = 0.05 (two-sided) comparing Hct (professional prick) vs. Hct (hematology analyzer).

Additionally the validation study of the StatStrip Hb/Hct device done by Nova Biomedical in Jan 2018 shows a lack in numbers of low (<35%) and high Hct values (>50%) from patients (capillary blood). Therefore, the current study also aims to include additional patients with low and high Hct to collect a broader range of Hct values for comparing accuracy of the device versus standard hematology analyzer. It is expected to enroll 60 patients within 6 months with following conditions:

- At least 60 % patients diagnosed with PV (WHO 2016)
- 30 % of patients with anemic conditions (Hct <35%) and
- 10 % patients with any other condition (Hct >50%)

Patients will be treated according to the clinical routine; schedule of visits will be decided therefore in the context of routine clinical practice. The patient will be informed of the nature of the study and will be asked to take part. The acceptance to take part of the study means that when the patient come to regular Hct controls, finger pricks with the StatStrip Xpress device will be done in parallel of the planed routine controls. At least one visit per patient (Baseline/Visit 1) will be carried out and documented in this study. If the patient comes again to the clinic for Hct testing in additional routine follow-up visits, the measurement with the device (finger prick) will be repeated equivalent to visit 1 and values documented in the eCRF. Patients can at any moment withdraw their informed consent to participate in the study. It is planned to document in total 60 visits in the eCRF within 6 months after study start to obtain 60 measurement points of Hct and Hb for the whole study. An interim analysis after approximately 3 months will show how many measuring points (and in which level: high, middle or low) are still needed to achieve enough precision and value range to perform a meaningful analyses based on Bland and Altman plots (mean vs. difference of measurements during each visit) and 95% confidence intervals for the mean difference (bias). Based on this assessment the need of further patient recruitment and data collection in the eCRF will be decided. There is a possibility that we collect 60 measurements but with a lower number of patients than anticipated for recruitment.

Patient data will be collected and entered into the study database after approvals from Ethics Committee and Health Authorities have been obtained and following the signature of informed consent by the patient.

4.2 Setting

4.2.1 Patient population

This observational study will include adult male and female patients diagnosed with a hematological condition and therefore visit the clinic to check Hct and/or Hb values in clinical routine obtained by the standard hematology analyzer. Please see inclusion criteria for details.

The patient will be informed about the possibility of participating in the study and only patients with a signed consent form can be included in the study. Patients shall be managed at the discretion of the treating physician following the local treatment recommendations which is based on the values obtained by the hematology analyzer and is not impaired by the outcome or values of the StatStrip Xpress 2 Hb/Hct device.

4.2.2 Inclusion criteria

Patients must meet all of the criteria below:

- Signed informed consent form
- Male or female patient of ≥ 18 years age

- Patients that visit the clinic and need a hematological check of Hct and/or Hb in normal clinical routine. This can be one of the following condition:
    a) patients with confirmed diagnosis of polycythemia vera (according to WHO criteria 2016)
    b) patients under anemic condition (Hct <35%)
    c) patients with any other condition (Hct > 50%)

4.2.3 Exclusion criteria

Patients must not meet any of the criteria below:

- Patients not willing to provide informed consent
- Patients for which a Hct and/or Hb measurement is not foreseen in the clinical routine
- Patients below 18 years of age
- Patients not willing to have a finger-prick and/or not willing to do the blood self-testing via StatStrip Xpress 2 Hb/Hct device
- Patients not able to do the blood self-testing via StatStrip Xpress 2 Hb/Hct device alone and have no care giver to do it for them

The primary reason should be provided if patient declines study participation or is not able to participate.

4.2.4 Criteria for premature patient withdrawal

Patients may voluntarily withdraw from the study or be dropped from the study at the discretion of the physician at any time.

Patients may discontinue from the study for the following reasons:

- Lost to follow-up by the center after signing informed consent
- Withdrawal of patient consent
- Study terminated by the sponsor
- Investigator decision

The primary reason for early discontinuation should be provided.

4.2.5 Initiation of study sites

During the initiation visit of the study site the StatStrip Xpress 2 Hb/Hct devices will undergo a validation test undertaken by the device manufacturer Nova Biomedical to assure the functionality of the device before using it during the study. (Nova Biomedical Corporation, Nov 2018 “Blood Hemoglobin and Hematocrit Monitoring System Clinical Analytical Evaluation Protocol”)

The device manufacturer Nova Biomedical will train the site personnel on correct blood sampling with the StatStrip Xpress 2 Hb/Hct device and site personnel will familiarize themselves with the content of the instructions for use.

Known sources of errors amongst other things are:

- uncleaned finger (fatty, dirty)
- wrong type of disinfectant that interferes with the measurement and strip reagent
- cold hands
- pressing the finger too much to get a blood drop
- no disposal of the first blood drop
- waiting too long until filling the strip (blood already started to agglutinate on the finger)

4.2.6 Assessment types

4.2.6.1 Blood sampling and measurement of Hct and Hb

The method of Hb/Hct measurement using the StatStrip Xpress 2 Hb/Hct device and its test strip is briefly described as follows:
Erythrocytes are hemolyzed to release Hb from the cells and Hb is oxidized by a reagent. The oxidation process induces electricity on the surface of the electrode. The current is dependent on the Hb concentration in the blood sample. The hematocrit is determined by a step by step impedance measurement method. The StatStrip Xpress 2 Hb/Hct device measures the current and impedance, calculates Hb concentration and Hct and displays the results on the device screen. For detailed information, see "Instructions for Use" (Nova StatStrip Xpress®2 Hämoglobin- und Hämatokrit-Messgerät für den Krankenhausgebrauch, Gebrauchsanweisung, Nova Biomedical Coporation, 2019).

No mandatory visit will be scheduled. Blood sampling will be done during a visit that has the aim to obtain Hct/Hb values in clinical routine. All participating study sites use venous blood samples in clinical routine for determining Hct/Hb on a hematology analyzer in the local lab on study site. During this study only the blood values from the hematology analyzer will be used for the treatment decision. The values from the StatStrip Xpress 2 Hb/Hct device will not contribute to it.

The consumables for the measurement with the StatStrip Xpress 2 Hb/Hct device are the following: Test strips for Hct/Hb (NovaBiomedical) and 21 gauge Single Use Safety Lancets

All assessments will be undertaken in the clinic. Nor the device neither the questionnaire are taken at home by the patient. The assessments and their order are described in Figure 1.

<u>Baseline</u>:

The Hct result from hematology analyzer is part of the evaluation of the inclusion criteria for the study. After confirming the eligibility of a patient by study personnel, the patient will be informed on the nature of the study and will be asked to take part.

The blood parameters from hematology analyzer of the former visit will be therefore retrospectively documented.

Visit 1

For the test of patient feasibility, it is essential to evaluate self-measurement with the StatStrip Xpress 2 Hb/Hct device with only minimal supervision. The patient will get the device and the strips including instructions for use and needs to do the finger-prick measurement on her/his own. The study personnel are available if main questions arise but is not allowed to show or explain the patient the procedure.

The following assessments will be undertaken in visit 1 and possible subsequent visits. The order of step 1. to 3. is mandatory but venous blood sample can be obtained before step 1. or after step 3.:

1. Patient self-prick: capillary blood sample with StatStrip Xpress 2 Hb/Hct device taken by the patient under minimal supervision
2. Professional prick: capillary blood sample of patient with StatStrip Xpress 2 Hb/Hct device taken by a trained study personnel

Note: The same finger prick can be used to obtain several blood drops if blood flow allows it; disposal of any agglutinated drop is then mandatory.

3. Patient is asked to fill out a patient questionnaire concerning handling and opinion of the StatStrip Xpress 2 Hb/Hct device

- Venous blood sample for the hematology analyzer

As a precaution, a cleaning procedure will be implemented after each StatStrip Xpress 2 Hb/Hct device is used (see study document “StatStrip Xpress 2 Hct/Hb Meter Cleaning Procedure”).

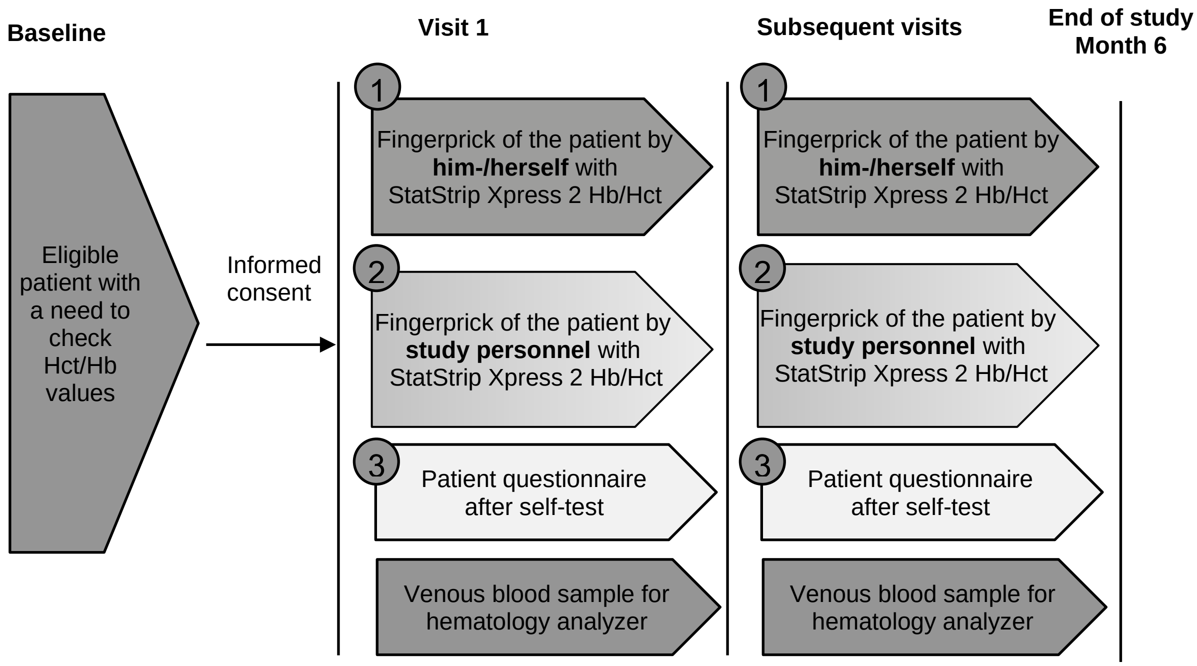

Figure 1: Visit scheme with focus on assessments and their order.

4.2.6.2 Patient and Physician questionnaire

Patients are asked to fill out a questionnaire (approx. 15 min) at each visit concerning the user friendliness, readability and understanding of the instructions for use and their opinion about the device. The questions are aligned with the device manufacturer Nova Biomedical and the technical wording are a requirement to support patient approval.

Study personnel will also fill out a different questionnaire (5 questions) at the end of the study to wrap up their opinion on handling the StatStrip Xpress 2 Hb/Hct device.

4.3 Data sources and Visit schedule

The data for this study will be documented in a standardized patient form retrieved from the sites and Novartis will support with the analysis.

Medical history/current medical conditions will be coded using the Medical Dictionary for Regulatory Activities (MedDRA) terminology.

Data on subjects collected on the StatStrip Xpress 2 Hb/Hct device during the study will be recorded in an anonymous fashion to ensure patient confidentiality. The physician must

maintain source documents where relevant for each patient in the study, consisting of all demographic and medical information, and keep the signed informed consent form (ICF) or an equivalent document. All information on CRFs must be traceable to these source documents in the patient's medical file.

Data collection schedule

There are no mandated visits in this study. Patients will be treated according to the routine medical practice and only these data will be collected in the study. These data include:

- Date of signature of Patient Informed Consent
- Date of the visit
- Underlying Disease/ Condition (that leads patient come to the clinic to check Hb and/or Hct)
- Date of diagnosis
- Year of birth
- ECOG performance status
- Hct and Hb (Hematology Analyzer, StatStrip Xpress 2 Hb/Hct device)
- RBC, WBC, platelets count
- Patient questionnaire to evaluate user friendliness of the device after each visit
- Study personnel questionnaire to evaluate user friendliness of the device
- **PV patients only:**
  - Treatment history of the last 12 months and actual treatments (cytoreductive treatment, anticoagulation treatment, phlebotomy)
  - Dates of study site visits of the last 12 months and corresponding Hct values

The below visit schedule is a recommendation for study site personnel and not mandatory.

Table 4-3-1 Visit schedule recommendation

Visits	Baseline	Visit 1	Possible subsequent visits	End of study (month 6)
Patient Informed Consent	x			
Condition / Disease		x		
Date of diagnosis		x		
Year of birth		x		
ECOG performance status		x	x	
hematology analyzer: Hb and Hct	x	x	x	
StatStrip Xpress 2 Hb/Hct device - Hb and Hct taken by:<br>Study personnel<br>Patient		x<br>x	x<br>x	
Patient questionnaire to evaluate the device		x	x	
Study personnel questionnaire				x
hematology analyzer: WBC, platelets, RBC		x	x	
**For PV patients only:**				
Cytoreductive treatment, anticoagulation treatment, phlebotomy:<br>treatment history of the last 12 months and<br>actual treatments		x	x	
Dates of all study site visits of the last 12 months and corresponding Hct values		x		

4.4 Data management

Patient data will be pseudonymized (patient number) and entered in an eCRF based on the system Studymate Pro. Definition of patient numbering: 3-digit number which is combination of 1-digit site number and a sequential 2-digit patient number (e.g. site 1, patient 1 = 101).

Data about study subjects will be kept confidential and managed under the applicable laws and regulations. Those regulations require a signed subject authorization informing the subject of the following:

- What protected health information (PHI) will be collected from subjects in this study;
- Who will have access to that information and why;
- Who will use or disclose that information;
- The rights of a research subject to revoke their authorization for use of their PHI.

In the event that a subject revokes authorization to collect or use PHI, the physician, by regulation, retains the ability to use all information collected prior to the revocation of subject authorization.

4.5 Data analysis

All statistical analyses will be performed under the direction of the CRO Mag. Andreas RAFFEINER GmbH personnel with support by the protocol authors. It is planned that the data from all centers that participate in this protocol will be used.

The data will be summarized with respect to demographic and baseline characteristics as well as blood values using descriptive statistics (quantitative data) and contingency tables (qualitative data).

Continuous variables will be summarized by number of patients (n), mean, standard deviation, minimum, median and maximum. Categorical variables will be summarized by absolute and relative frequencies. The number and percentage of patients in each category will be presented for categorical variables for all patients.

4.5.1 Analysis set

Consists of all patients enrolled in the study.

4.5.2 Analysis of variables

All comparisons between different methods as there are:

- Hct (professional prick) vs. Hct (hematology analyzer)
- Hct (patient self-prick) vs. Hct (hematology analyzer)
- Hct (patient self-prick) vs. Hct (professional prick)

➔ same comparisons will be done for Hb values

will be done using the Bland and Altman plot as well as one sample t-test for equivalence, testing the mean difference for not exceeding 1 percentage point in Hct and 1 g/dL in Hb.

Correlation coefficients (Pearson and Spearman) as well as scatter plots will be used to investigate possible impact of a high blood cell count (RBC, WBC, platelet count) on Hct values obtained by professional prick.

All other variables will be analyzed in a descriptive manner.

4.5.3 Handling of missing values

Missing values will not be replaced. Every effort will be made by the physician to collect all data available through the routine monitoring of the patients. Due to the non-interventional nature of the study no effort will be made to repeat missed examinations for the purpose of this

protocol, unless these are part of the routine treatment of the patients. Counts of missing values for both continuous and categorical variables will be reported.

4.6 Quality control - Data recording and document retention

In all scenarios, the physician must maintain source documents for each patient in the study, consisting of case and visit notes (hospital or clinic medical records) containing demographic and medical information, and the results of any other tests or assessments. All data entered in the study database must be traceable to these source documents in the patient’s file.

5 Protection of human subjects

The protocol, the questionnaires and the proposed ICF must be reviewed and approved by a constituted Ethics Committee (EC) before study start.

5.1 Informed consent procedures

The physician must keep the original ICF signed by the patient (a signed copy is given to the patient).

Eligible patients may only be included in the study after providing written (witnessed, where required by law or regulation), EC approved informed consent. If the patient is capable of doing so, he/she should assent by personally signing and dating the written informed consent document or a separate assent form. The process of obtaining informed consent should be documented in the patient source documents.

6 Management and reporting of adverse events/adverse reactions

No solicited safety data capture is required for studies involving primary data collection without a Novartis drug of interest.

However, if during the course of the study, an **adverse reaction** (i.e. an adverse event suspected to be associated with the use of a drug) is identified in a patient receiving a Novartis product, it must be reported to Novartis as a spontaneous report or to the local Health Authority if required by local regulatory requirements for individual case safety reporting.

Adverse reactions identified for the StatStrip Xpress 2 Hb/Hct device and its consumables should be reported to Nova Biomedical Corporation (Medical & Scientific Affairs, 200 Prospect Street,Waltham, MA 02454-9141, USA; Mobile:+49-172-4371732, Mail: adeutsch@novabio.com).

Adverse reactions identified for all other products should be reported to the local Health Authority in accordance with national regulatory requirements for individual case safety reporting or the Marketing Authorization Holder.

7 Plans of disseminating and communicating study results

Upon study completion and finalization of the study report, the results of this study may be either submitted for publication and/or posted in a publicly accessible database of results. Publications will comply with the International Committee of Medical Journal Editors (ICMJE) guidelines.

8 References

Arber DA, Orazi A, Hasserjian R et al. (2016) The 2016 revision to the World Health Organization classification of myeloid neoplasms and acute leukemia. Blood. 127(20):2391-405

Barbui T, Thiele J, Gisslinger H et al. (2018) The 2016 WHO classification and diagnostic criteria for myeloproliferative neoplasms: document summary and in-depth discussion. Blood Cancer J. 8(2):15.

Barosi G, Mesa R, Finazzi G, et al. (2013) Revised response criteria for polycythemia vera and essential thrombocythemia: a ELN and IWG-MRT consensus project. Blood; 121(23): 4778-4781.

Basch E, Deal AM, Kris MG, et al. (2016) Symptom Monitoring With Patient-Reported Outcomes During Routine Cancer Treatment: A Randomized Controlled Trial. J Clin Oncol. 34(6):557-65.

Basch E, Deal AM, Dueck AC et al. (2017) Overall Survival Results of a Trial Assessing Patient-Reported Outcomes for Symptom Monitoring During Routine Cancer Treatment. JAMA July 11, 2017 Volume 318, Number 2

Berk PD, Goldberg JD, Donovan PB, Fruchtman SM, Berlin NI, Wasserman LR. (1986) Therapeutic recommendations in polycythemia vera based on Polycythemia Vera Study Group protocols. Semin Hematol; 23(2):132-143.

Clinical & Laboratory Standards Institute (CLSI). (2018) Measurement Procedure Comparison and Bias Estimation Using Patient Samples – 3rd Edition. EP9c

da Silva Saraiva S, Orsi FA, Santos MP et al. (2016) Home management of INR in the public health system: feasibility of self-management of oral anticoagulation and long-term performance of individual POC devices in determining INR. J Thromb Thrombolysis. 42(1):146-53.

Declaration of Helsinki (https://www.wma.net/policies-post/wma-declaration-of-helsinki-ethical-principles-for-medical-research-involving-human-subjects)

Denis F, Lethrosne C, Pourel N, et al. (2017) Randomized Trial Comparing a Web-Mediated Follow-up With Routine Surveillance in Lung Cancer Patients. J Natl Cancer Inst. 109(9).

Denis F, Basch E, Septans AL, et al. (2019) Two-Year Survival Comparing Web-Based Symptom Monitoring vs Routine Surveillance Following Treatment for Lung Cancer. JAMA; 321(3): 306–7.

Human Research Act (HRA): http://www.admin.ch/opc/en/classified-compilation/20121176/201401010000/810.305.pdf

Landolfi, R. et al. (2004) Efficacy and safety of low-dose aspirin in polycythemia vera. N. Engl. J. Med. 350, 114–124

Marchioli R, Finazzi G, Specchia G, et al. (2013) Cardiovascular events and intensity of treatment in polycythemia vera. N Eng J Med; 368(1):22-33.

Mesa RA, Niblack J, Wadleigh M, et al. (2007) The burden of fatigue and quality of life in myeloproliferative disorders (MPDs). an international Internet-based survey of 1179 MPD patients. Cancer; 109(1):68-76.

Nova Biomedical Corporation (Jan 2018) StatStrip® Hemoglobin and Hematocrit Meter System, Analytical Performance Testing

Nova Biomedical Coporation (2019) Nova StatStrip Xpress®2 Hämoglobin- und Hämatokrit-Messgerät für den Krankenhausgebrauch, Gebrauchsanweisung

Ordinance on Human Research with the Exception of Clinical trials (HRO) https://www.admin.ch/opc/en/classified-compilation/20121177/index.html

Schnell O, Alawi H, Battelino T et al. (2011) Addressing schemes of self-monitoring of blood glucose in type 2 diabetes: a European perspective and expert recommendation. Diabetes Technol Ther. 13(9):959-65.

Stuart BJ, Viera AJ. Polycythemia vera. (2004) Am Fam Physician; 69(9):2139-2144.

Tefferi A. and Barbui T. (2017) Polycythemia vera and essential thrombocythemia: 2017 update on diagnosis, risk-stratification, and management. Am J Hematol.92(1):94-108
